# Trends in Cholera Epidemiology

**DOI:** 10.1371/journal.pmed.0030042

**Published:** 2006-01-31

**Authors:** Claudia T Codeço, Flavio C Coelho

## Abstract

Codeço and Coelho discuss a new study on cholera modeling that helps us to better explain the observed epidemic pattern of the disease.

Cholera has been scrutinized since the birth of epidemiology, and it is still a subject of intense interest for modern-day epidemiologists. Studying cholera has led to the development of new epidemiological methods that have helped to illuminate not only cholera transmission but the whole science of infectious disease epidemiology.

It was John Snow in London in the 1800s who originally established a causal link between cholera transmission and exposure to contaminated water ( Figure 1). His work on cholera was fundamental in many ways: he proposed methods and ideas that are still part of the basic toolkit of modern epidemiology, such as time–spatial analysis and notions of source of exposure and incubation periods [ 1]. More recently, researchers have begun to understand more about the mechanisms of infectiousness of the cholera pathogen Vibrio cholerae. And in a new study in *PLoS Medicine*, David Hartley and colleagues have adjusted existing approaches to modeling cholera to evaluate how these recently found mechanisms of infectiousness can help us better explain the observed epidemic pattern of the disease [ 2].

**Figure 1:**
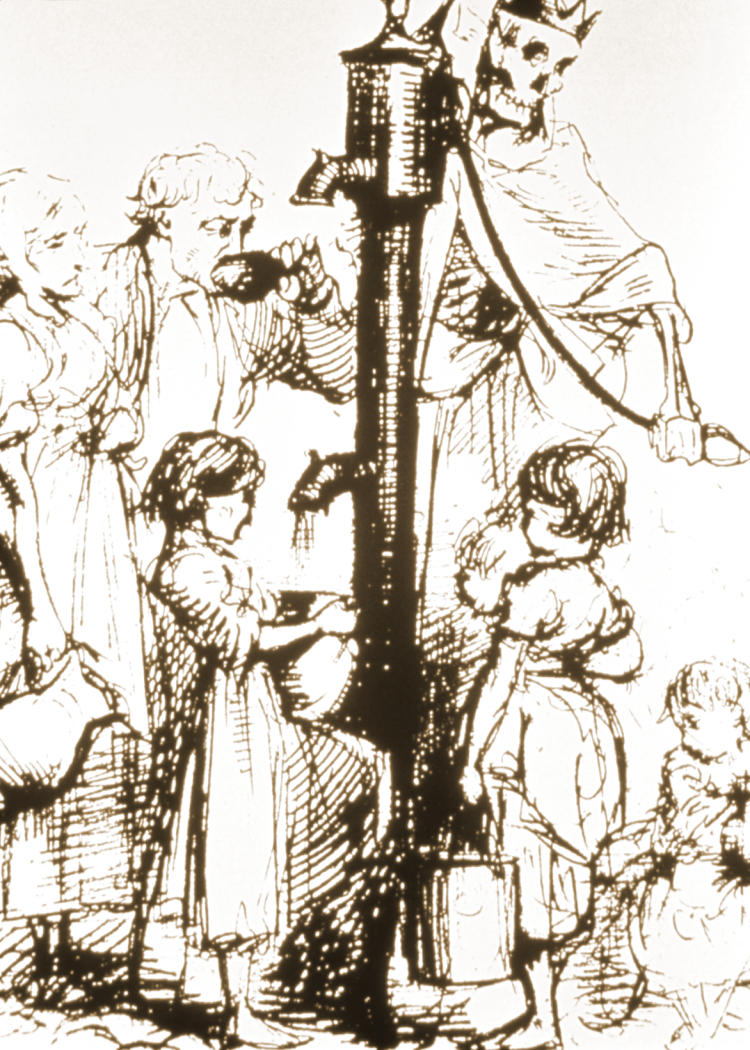
“Death's Dispensary” This sketch was drawn in 1866, around the same time that John Snow published his definitive studies on cholera transmission. The contaminated water supply of London, like that of other major European capitals, was untreated river water. (Illustration: John Pinwell)

## Cholera Outbreaks: Two Puzzling Features

Cholera is caused by the toxin-producing bacterium V. cholerae. In endemic regions, such as South Asia, cholera is seasonal, with explosive outbreaks occurring once or twice a year, depending on the region. Periodically, pandemic waves of cholera roll across the world causing a heavy death toll.

Two features of cholera outbreaks are puzzling: their almost simultaneous appearance in distinct areas (suggesting an environmental trigger) and their explosive nature. Until the 1970s, V. cholerae was thought to be a human-specialized parasite, incapable of persisting outside its host. But in the 1990s, it became clear that V. cholerae was a successful member of the brackish water microbial community, living in association with plankton in an unculturable but viable state [ 3]. This finding sparked a debate on the relative importance of human-to-human transmission versus transmission from environment to humans. The finding that V. cholerae lived in brackish water shifted the balance toward the environmental hypothesis, that is, the hypothesis that seasonal outbreaks are triggered by seasonal blooming of aquatic V. cholerae [ 4].

But the other aspect of cholera outbreaks—its explosive nature—was still unexplained. Volunteer studies suggest that cholera infection requires consumption of a heavy infectious dose, which is unlikely to be found in the environment in the beginning of the epidemic season, even considering the blooming of aquatic V. cholerae. An important part of the puzzle appeared to be missing.

## The Hyperinfectious State

In 2002, Merrell and colleagues proposed an audacious hypothesis, based on a set of competition experiments in infant mice [ 5]. They fed mice with mixed cultures of V. cholerae recently isolated from the stools of human patients and V. cholerae grown in vitro, and then observed which of these bacteria were more successful colonizers of the mouse small intestine. They found that, in all replicates of the experiment, the stool-derived bacteria were always more successful than the lab-cultured bacteria in colonizing the gut, reaching ratios as large as 700 stool-derived bacteria to one cultured bacterium. Their interpretation of this result was that the passage of V. cholerae through the human gut would promote the expression of genes that would make the bacteria more infective, that is, more capable of surviving and growing in the intestinal environment. Interestingly, this “hyperinfectious state,” as they called it, would be lost after a few hours outside the gut—that is, after being outside the gut for more than 18 hours, the competitive advantage of stool bacteria vanishes.

But what would be the epidemic impact of such a transient behavior? This was the question posed by David Hartley and colleagues [ 2]. It is the type of question that mathematical models are well suited to answer.

A mathematical model, in this case, is a quantitative representation of the mechanisms that we think are important for the spread of the disease. Quantification allows us to use mathematical theorems to derive the behavior of the system based on the mechanisms proposed. In terms of epidemiology, it means that we can use a model to evaluate how a set of mechanisms would translate into a dynamic behavior.

Hartley and colleagues proposed a mechanistic model for cholera transmission with two bacterial states: hyperinfectious (HI) and non-HI. The infectious dose is 700 times smaller if consumed water contains HI bacteria. Infected persons shed HI bacteria into their feces, which decay to the non-HI state in an average of 18 hours. Using this model, they derived an expression for the basic reproductive number, which is a measure of the velocity of epidemic spread. This expression is the product of two terms: the number of cases produced by contact with recently shed bacteria (“direct or quasidirect transmission”) and the number of cases produced by contact with non-HI bacteria (“indirect transmission”). If both modes of transmission are equally important in a community, explosive outbreaks are expected. This would be the case in a community where poor basic hygienic conditions make contact with recently shed bacteria a probable event.

## Public Health Implications

One of the main implications of Hartley and colleagues' study is that any public health action to reduce direct transmission will have a large impact on the rate of disease spread. In other words, any measure that delays fecal–oral transmission, even the simple act of washing hands before a meal, would have a stronger than previously expected impact on cholera transmission. Thus, public health strategies based on increasing hygiene standards would be effective, even if more permanent improvements, such as proper sewage treatment, were impossible. The balance therefore shifts back to the importance of human-to-human transmission.

But the presence of the hyperinfective state is still poorly understood, and more studies are required to better understand its prevalence and mechanisms, and how it changes our current estimates of infectious dosages for cholera. It seems that, at least for the near future, cholera will maintain its role as a catalyst for new ideas about infectious disease transmission.

## Abbreviation

HI: hyperinfectious
